# Crystal structure of (*E*)-13-(pyrimidin-5-yl)parthenolide

**DOI:** 10.1107/S2056989015021507

**Published:** 2015-11-28

**Authors:** Shobanbabu Bommagani, Narsimha R. Penthala, Sean Parkin, Peter A. Crooks

**Affiliations:** a Dept. of Pharm. Sciences, College of Pharmacy, University of Arkansas for Medical Sciences, Little Rock, AR 72205, USA; b Dept. of Chemistry, University of Kentucky, Lexington KY 40506, USA

**Keywords:** crystal structure, parthenolide, pyrimidine, Heck product

## Abstract

The title compound, C_19_H_22_N_2_O_3_, {systematic name (1a*R*,4*E*,7a*S*,8*E*,10a*S*,10b*R*)-1a,5-dimethyl-8-[(pyrimidin-5-yl)­methylid­ene]-2,3,6,7,7a,8,10a,10b-octa­hydro­oxireno[2′,3′:9,10]cyclo­deca­[1,2-*b*]furan-9(1a*H*)-one} was obtained from the reaction of parthenolide [systematic name (1a*R*,7a*S*,10a*S*,10b*R*,*E*)-1a,5-dimethyl-8-methyl­ene-2,3,6,7,7a,8,10a,10b-octa­hydro­oxireno[2′,3′:9,10]cyclodeca­[1,2-*b*]furan-9(1a*H*)-one] with 5-bromo­pyrimidine under Heck reaction conditions, and was identified as an *E* isomer. The mol­ecule possesses ten-, five- (lactone) and three-membered (epoxide) rings with a pyrimidine group as a substituent. The ten-membered ring displays an approximate chair–chair conformation, while the lactone ring shows a flattened envelope-type conformation. The dihedral angle between the pyrimidine moiety and the lactone ring system is 29.43 (7)°.

## Abstract 2

The title mol­ecule possesses ten-, five- (lactone) and three-membered (epoxide) rings with a pyrimidine group as a substituent. The ten-membered ring displays an approximate chair–chair conformation, while the lactone ring shows a flattened envelope-type conformation.

## Chemical context   

Parthenolide (PTL) is a sesquiterpene lactone known to significantly target cancer stem cells, which are the putative roots of all types of cancer (Gopal *et al.*, 2007 ▸). PTL has been isolated from several different plant species, feverfew leaf (*Tanacetum parthenium*) being one of the major sources (Awang, 1989 ▸). PTL exhibits a wide range of biological activities, such as anti-inflammatory, anti-bacterial, anti-fungal, and cytotoxic properties (Picman, 1986 ▸). Consequently, PTL was discovered to be capable of inducing robust apoptosis in primary acute myelogenous leukemia (AML) cells (Guzman *et al.*, 2007 ▸), proving to be equally effective among all subpopulations within primary AML specimens, including leukemia stem cells (LSCs). Gopal *et al.* (2007 ▸) reported that PTL specifically depletes HDAC1 protein without affecting other class I/II HDACs (histone de­acetyl­ases). Nasim *et al.* (2008 ▸) reported the anti-leukemic activity of amino­parthenolide analogues. Han *et al.* (2009 ▸) reported on bioactive derivatives of Heck products of PTL. Recently, Penthala *et al.* (2014*a* ▸) reported the anti-cancer activity of PTL–Heck products. Recently we (Penthala *et al.*, 2014*b* ▸) reported the crystal structure of 13-{4-[*Z*–2-cyano-2-(3,4,5-tri­meth­oxy­phen­yl)ethen­yl]phen­yl} parthenolide, an analog of PTL, which was found to have the *E* configuration at C-13. The inter­esting biological properties of PTL directed our attention to design and synthesize additional bioactive derivatives. In order to obtain detailed information on the structural conformation of the current mol­ecule, including assignment of the absolute configuration of the four stereocentres, and to establish the geometry of the exocyclic double bond, a single crystal X-ray structure determination has been carried out.
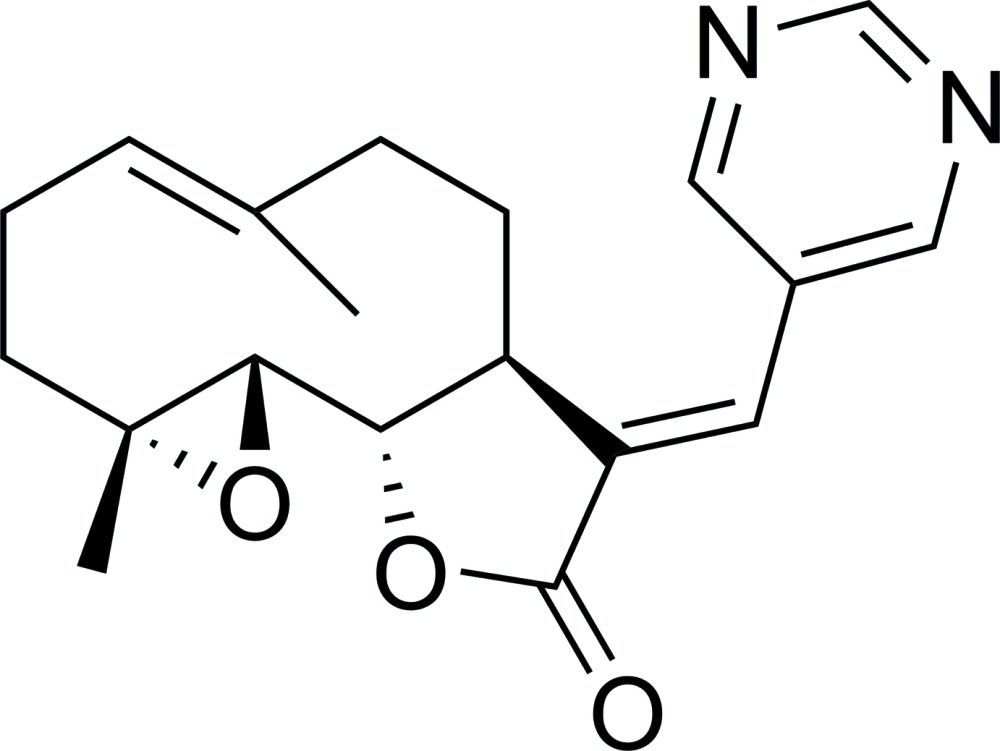


## Structural commentary   

**Figure 1:**
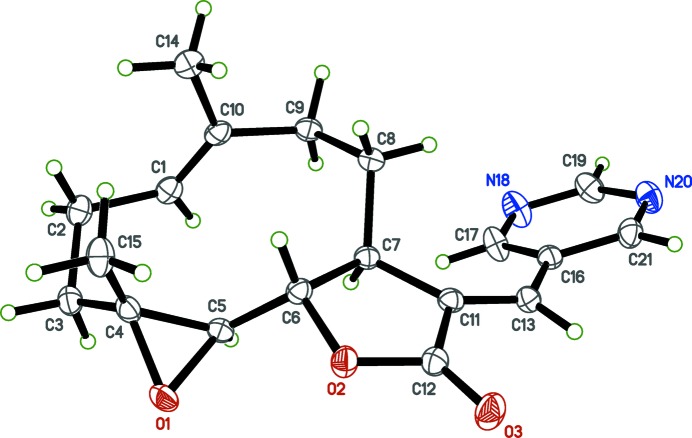
The mol­ecular structure of the title compound with probability ellipsoids drawn at the 50% probability level.

The title compound is shown in Fig. 1 ▸. The PTL substructure of the mol­ecule contains a ten-membered carbocyclic ring (chair–chair conformation) fused to a lactone ring (flattened envelope-type conformation), and an epoxide ring, as previously reported (Castañeda-Acosta & Fisher, 1993 ▸). The title compound contains an *E*-exocyclic olefinic bond C11=C13. The pyrimidine ring is twisted out of the plane of the furan ring, making a dihedral angle of 29.43 (7)°. The C11=C13—C16 bond angle of 127.89 (16)° deviates from the ideal value of 120°, but other bond lengths and angles are largely unremarkable. The four chiral carbon atoms in PTL were determined using 1354 quotients (Parsons *et al.*, 2013 ▸) as follows: C4(*R*),C5(*R*),C6(*S*),C7(*S*) for the arbitrary atom-numbering scheme used, and is consistent with previous studies (Penthala *et al.*, 2013 ▸).

## Supra­molecular features   

There are no classical hydrogen bonds and no π–π inter­actions. There are a few C—H⋯N and C—H⋯O short contacts, but none that have the right geometry to be considered as non-classical hydrogen bonds. Inter­molecular contacts thus appear to be limited to van der Waals inter­actions.

## Database survey   

A search of the November 2014 release of the Cambridge Structure Database (Groom & Allen, 2014 ▸) for the PTL substructure gave 24 hits. Two of these (PARTEN: Quick & Rogers, 1976 ▸; PARTEN01: Bartsch *et al.*, 1983 ▸) give the structure of PTL itself, with the remaining 22 being substituted variants of PTL. Of these substituted parthenolides, only four CSD entries: HORZOF (Penthala *et al.*, 2014*b* ▸), HUKLAB, HUKLEF (Han *et al.*, 2009 ▸) and QILGEZ (Penthala *et al.*, 2013 ▸), are substituted at the exocyclic double bond.

## Synthesis and crystallization   

**Synthetic procedures:** The title compound, containing the PTL substructure, was synthesized by the previously reported literature procedure (Han *et al.*, 2009 ▸). In brief, parthenolide (1 mmol), 5-bromo­pyrimidine (1.1 mmol), tri­ethyl­amine (3.0 mmol) and 5 mol% of palladium acetate were charged into di­methyl­formamide (2 ml) at room temperature. The reactants were stirred at 333–343 K for 24 h. After completion of the reaction, the reaction mass was extracted into diethyl ether (2 × 30 ml). The combined organic layers were dried over anhydrous sodium sulfate, concentrated and purified by column chromatography. The title compound was recrystallized from a mixture of hexane and acetone (9:1), which gave colourless needles upon slow evaporation of the solution at room temperature over 24 h.

## Refinement   

Crystal data, data collection and structure refinement details are summarized in Table 1 ▸. H atoms were found in difference Fourier maps, but subsequently included in the refinement using riding models, with constrained distances set to 0.95 Å (C*sp* ^2^H), 0.98 Å (*R*CH_3_), 0.99 Å (*R*_2_CH_2_) and 1.00 Å (*R*_3_CH). *U*_iso_(H) parameters were set to values of either 1.2*U*_eq_ or 1.5*U*_eq_ (*R*CH_3_ only) of the attached atom. The absolute structure parameter [−0.04 (3)] was determined directly from the diffraction data using 1354 Parsons quotients (Parsons *et al.*, 2013 ▸), with the four chiral carbon atoms assigned to be *R*,*R*,*S*,*S* for the arbitrarily numbered atoms C4, C5, C6, C7, respectively.

Refinement progress was checked using *PLATON* (Spek, 2009 ▸) and by an *R*-tensor (Parkin, 2000 ▸). 

## Supplementary Material

Crystal structure: contains datablock(s) I, global. DOI: 10.1107/S2056989015021507/zs2350sup1.cif

Structure factors: contains datablock(s) I. DOI: 10.1107/S2056989015021507/zs2350Isup2.hkl

CCDC reference: 1436825

Additional supporting information:  crystallographic information; 3D view; checkCIF report

## Figures and Tables

**Table 1:** Experimental details

Crystal data	
Chemical formula	C_19_H_22_N_2_O_3_
*M*_r_	326.38
Crystal system, space group	Monoclinic, *P*2_1_
Temperature (K)	90
*a*, *b*, *c* (Å)	10.3526 (2), 7.2612 (1), 11.9198 (2)
β (°)	108.1210 (6)
*V* (Å^3^)	851.60 (2)
*Z*	2
Radiation type	Cu *K*α
μ (mm^−1^)	0.70
Crystal size (mm)	0.25 × 0.13 × 0.10

Data collection	
Diffractometer	Bruker X8 Proteum
Absorption correction	Multi-scan (*SADABS*; Bruker, 2006 ▸)
*T*_min_, *T*_max_	0.850, 0.942
No. of measured, independent and observed [*I* > 2σ(*I*)] reflections	11370, 3020, 3013
*R*_int_	0.032
(sin θ/λ)_max_ (Å^−1^)	0.602

Refinement	
*R*[*F* ^2^ > 2σ(*F* ^2^)], *wR*(*F* ^2^), *S*	0.027, 0.068, 1.05
No. of reflections	3020
No. of parameters	220
No. of restraints	1
H-atom treatment	H-atom parameters constrained
Δρ_max_, Δρ_min_ (e Å^−3^)	0.15, −0.14
Absolute structure	Flack *x* determined using 1354 quotients [(*I* ^+^)−(*I* ^−^)]/[(*I* ^+^)+(*I* ^−^)] (Parsons *et al.*, 2013 ▸).
Absolute structure parameter	−0.04 (3)

Computer programs: *APEX2* and *SAINT* (Bruker, 2006 ▸), *SHELXS97*, *SHELXTL* and *XP* in *SHELXTL* (Sheldrick, 2008 ▸), *SHELXL2014* (Sheldrick, 2015 ▸) and *CIFFIX* (Parkin, 2013 ▸).

## supplementary crystallographic information

### Crystal data

**Table:**

C_19_H_22_N_2_O_3_	*F*(000) = 348
*M**_r_* = 326.38	*D*_x_ = 1.273 Mg m^−^^3^
Monoclinic, *P*2_1_	Cu *K*α radiation, λ = 1.54178 Å
*a* = 10.3526 (2) Å	Cell parameters from 9908 reflections
*b* = 7.2612 (1) Å	θ = 4.5–68.2°
*c* = 11.9198 (2) Å	µ = 0.70 mm^−^^1^
β = 108.1210 (6)°	*T* = 90 K
*V* = 851.60 (2) Å^3^	Solvent-rounded block, colourless
*Z* = 2	0.25 × 0.13 × 0.10 mm

### Data collection

**Table:**

Bruker X8 Proteum diffractometer	3020 independent reflections
Radiation source: fine-focus rotating anode	3013 reflections with *I* > 2σ(*I*)
Detector resolution: 5.6 pixels mm^-1^	*R*_int_ = 0.032
φ and ω scans	θ_max_ = 68.2°, θ_min_ = 4.5°
Absorption correction: multi-scan (*SADABS*; Bruker, 2006)	*h* = −12→10
*T*_min_ = 0.850, *T*_max_ = 0.942	*k* = −8→8
11370 measured reflections	*l* = −14→13

### Refinement

**Table:**

Refinement on *F*^2^	Hydrogen site location: difference Fourier map
Least-squares matrix: full	H-atom parameters constrained
*R*[*F*^2^ > 2σ(*F*^2^)] = 0.027	*w* = 1/[σ^2^(*F*_o_^2^) + (0.0326*P*)^2^ + 0.1773*P*] where *P* = (*F*_o_^2^ + 2*F*_c_^2^)/3
*wR*(*F*^2^) = 0.068	(Δ/σ)_max_ < 0.001
*S* = 1.05	Δρ_max_ = 0.15 e Å^−^^3^
3020 reflections	Δρ_min_ = −0.14 e Å^−^^3^
220 parameters	Extinction correction: *SHELXL-2014*, Fc^*^=kFc[1+0.001xFc^2^λ^3^/sin(2θ)]^-1/4^
1 restraint	Extinction coefficient: 0.010 (2)
Primary atom site location: structure-invariant direct methods	Absolute structure: Flack x determined using 1354 quotients [(*I*^+^)-(*I*^-^)]/[(*I*^+^)+(*I*^-^)] (Parsons *et al.*, 2013).
Secondary atom site location: difference Fourier map	Absolute structure parameter: −0.04 (3)

### Special details

**Table:**

Experimental. The crystal was mounted with polyisobutene oil on the tip of a fine glass fibre, which was fastened in a copper mounting pin with electrical solder. It was placed directly into the cold gas stream of a liquid nitrogen based cryostat, according to published methods. Diffraction data were collected with the crystal at 90 K, which is standard practice in this laboratory for the majority of flash-cooled crystals.The crystals were large, and could not be cut to size without inducing damage by crushing, leading to shattered, frayed ends. These damaged parts could easily be dissolved away, however, to give solvent-rounded undamaged pieces of optimal size for data collection.
Geometry. All e.s.d.'s (except the e.s.d. in the dihedral angle between two l.s. planes) are estimated using the full covariance matrix. The cell e.s.d.'s are taken into account individually in the estimation of e.s.d.'s in distances, angles and torsion angles; correlations between e.s.d.'s in cell parameters are only used when they are defined by crystal symmetry. An approximate (isotropic) treatment of cell e.s.d.'s is used for estimating e.s.d.'s involving l.s. planes.
Refinement. Refinement progress was checked using *PLATON* (Spek, 2009) and by an *R*-tensor (Parkin, 2000). The final model was further checked with the IUCr utility *checkCIF*.

### Fractional atomic coordinates and isotropic or equivalent isotropic displacement parameters (Å^2^)

**Table:**

	*x*	*y*	*z*	*U*_iso_*/*U*_eq_	
O1	0.59428 (12)	0.24072 (19)	0.48957 (10)	0.0228 (3)	
O2	0.30633 (12)	0.25486 (17)	0.43887 (10)	0.0195 (3)	
O3	0.12397 (13)	0.29255 (19)	0.49855 (11)	0.0266 (3)	
C1	0.57679 (17)	0.5285 (2)	0.19945 (14)	0.0185 (4)	
H1A	0.5833	0.6439	0.2384	0.022*	
C2	0.70386 (17)	0.4134 (3)	0.23289 (16)	0.0208 (4)	
H2A	0.7809	0.4881	0.2257	0.025*	
H2B	0.6915	0.3076	0.1781	0.025*	
C3	0.73676 (17)	0.3417 (3)	0.36116 (15)	0.0202 (4)	
H3A	0.8146	0.2554	0.3792	0.024*	
H3B	0.7615	0.4460	0.4173	0.024*	
C4	0.61322 (17)	0.2447 (2)	0.37345 (14)	0.0178 (4)	
C5	0.51035 (16)	0.3615 (2)	0.40010 (13)	0.0165 (3)	
H5A	0.5353	0.4948	0.4118	0.020*	
C6	0.35977 (16)	0.3252 (2)	0.34753 (14)	0.0159 (3)	
H6A	0.3445	0.2321	0.2829	0.019*	
C7	0.27894 (16)	0.5036 (2)	0.29783 (13)	0.0149 (3)	
H7A	0.3396	0.6113	0.3292	0.018*	
C8	0.22800 (16)	0.5184 (3)	0.16174 (14)	0.0176 (4)	
H8A	0.1434	0.5923	0.1376	0.021*	
H8B	0.2058	0.3936	0.1278	0.021*	
C9	0.33322 (17)	0.6078 (3)	0.11062 (14)	0.0185 (4)	
H9A	0.2884	0.6358	0.0261	0.022*	
H9B	0.3638	0.7258	0.1519	0.022*	
C10	0.45591 (17)	0.4887 (2)	0.12176 (14)	0.0179 (4)	
C11	0.16862 (16)	0.5027 (2)	0.35605 (14)	0.0159 (3)	
C12	0.19100 (17)	0.3425 (3)	0.43718 (14)	0.0184 (4)	
C13	0.06867 (17)	0.6212 (3)	0.35248 (14)	0.0179 (4)	
H13A	0.0096	0.5872	0.3961	0.021*	
C14	0.42876 (19)	0.3267 (3)	0.03864 (16)	0.0269 (4)	
H14A	0.5140	0.2609	0.0473	0.040*	
H14B	0.3908	0.3705	−0.0428	0.040*	
H14C	0.3639	0.2434	0.0573	0.040*	
C15	0.57578 (18)	0.0643 (2)	0.30934 (18)	0.0239 (4)	
H15A	0.5012	0.0072	0.3311	0.036*	
H15B	0.6548	−0.0179	0.3312	0.036*	
H15C	0.5470	0.0861	0.2240	0.036*	
C16	0.03901 (16)	0.7977 (2)	0.28928 (14)	0.0180 (4)	
C17	0.13568 (18)	0.9097 (3)	0.26601 (18)	0.0244 (4)	
H17A	0.2281	0.8719	0.2933	0.029*	
N18	0.10538 (16)	1.0688 (2)	0.20684 (16)	0.0293 (4)	
C19	−0.02533 (19)	1.1156 (3)	0.17269 (17)	0.0252 (4)	
H19A	−0.0485	1.2265	0.1287	0.030*	
N20	−0.12697 (15)	1.0248 (2)	0.19312 (14)	0.0256 (4)	
C21	−0.09291 (18)	0.8673 (3)	0.25279 (15)	0.0219 (4)	
H21A	−0.1621	0.7994	0.2713	0.026*	

### Atomic displacement parameters (Å^2^)

**Table:**

	*U*^11^	*U*^22^	*U*^33^	*U*^12^	*U*^13^	*U*^23^
O1	0.0188 (6)	0.0306 (7)	0.0176 (6)	0.0061 (5)	0.0035 (5)	0.0092 (5)
O2	0.0187 (6)	0.0200 (6)	0.0221 (6)	0.0038 (5)	0.0098 (5)	0.0064 (5)
O3	0.0242 (6)	0.0305 (8)	0.0303 (7)	0.0031 (6)	0.0162 (5)	0.0097 (6)
C1	0.0218 (8)	0.0163 (8)	0.0197 (8)	−0.0006 (7)	0.0100 (7)	0.0024 (7)
C2	0.0184 (8)	0.0208 (9)	0.0254 (9)	−0.0018 (7)	0.0101 (7)	−0.0001 (7)
C3	0.0157 (8)	0.0216 (9)	0.0228 (8)	0.0016 (7)	0.0053 (6)	−0.0004 (7)
C4	0.0163 (8)	0.0184 (8)	0.0174 (8)	0.0036 (7)	0.0031 (6)	0.0040 (7)
C5	0.0175 (8)	0.0173 (8)	0.0141 (7)	0.0020 (7)	0.0043 (6)	0.0031 (6)
C6	0.0173 (8)	0.0162 (8)	0.0154 (7)	0.0002 (6)	0.0068 (6)	0.0012 (6)
C7	0.0155 (7)	0.0147 (8)	0.0142 (7)	−0.0011 (6)	0.0041 (6)	−0.0008 (6)
C8	0.0172 (8)	0.0201 (8)	0.0143 (8)	0.0005 (7)	0.0034 (6)	0.0003 (7)
C9	0.0209 (8)	0.0195 (8)	0.0147 (7)	0.0018 (7)	0.0051 (6)	0.0044 (7)
C10	0.0221 (8)	0.0190 (9)	0.0150 (7)	0.0003 (7)	0.0092 (7)	0.0026 (7)
C11	0.0153 (7)	0.0170 (8)	0.0143 (7)	−0.0021 (7)	0.0031 (6)	−0.0010 (7)
C12	0.0166 (8)	0.0206 (9)	0.0187 (8)	0.0004 (7)	0.0065 (6)	0.0014 (7)
C13	0.0144 (8)	0.0213 (9)	0.0175 (8)	−0.0011 (7)	0.0044 (6)	−0.0018 (7)
C14	0.0258 (9)	0.0321 (11)	0.0219 (8)	0.0041 (8)	0.0061 (7)	−0.0061 (8)
C15	0.0206 (8)	0.0177 (9)	0.0346 (10)	0.0023 (7)	0.0105 (8)	0.0005 (7)
C16	0.0175 (8)	0.0190 (9)	0.0161 (7)	0.0009 (7)	0.0032 (6)	−0.0044 (7)
C17	0.0156 (8)	0.0189 (9)	0.0350 (10)	0.0005 (7)	0.0024 (8)	0.0019 (8)
N18	0.0209 (8)	0.0216 (9)	0.0409 (10)	−0.0014 (6)	0.0030 (7)	0.0062 (7)
C19	0.0240 (9)	0.0183 (9)	0.0288 (9)	0.0021 (8)	0.0016 (7)	0.0019 (8)
N20	0.0206 (8)	0.0232 (8)	0.0304 (8)	0.0042 (6)	0.0043 (6)	0.0003 (7)
C21	0.0194 (8)	0.0219 (9)	0.0253 (9)	0.0026 (7)	0.0084 (7)	−0.0024 (7)

### Geometric parameters (Å, º)

**Table:**

O1—C5	1.444 (2)	C8—H8B	0.9900
O1—C4	1.457 (2)	C9—C10	1.508 (2)
O2—C12	1.348 (2)	C9—H9A	0.9900
O2—C6	1.4585 (19)	C9—H9B	0.9900
O3—C12	1.210 (2)	C10—C14	1.507 (3)
C1—C10	1.337 (2)	C11—C13	1.336 (2)
C1—C2	1.504 (2)	C11—C12	1.484 (2)
C1—H1A	0.9500	C13—C16	1.470 (2)
C2—C3	1.549 (2)	C13—H13A	0.9500
C2—H2A	0.9900	C14—H14A	0.9800
C2—H2B	0.9900	C14—H14B	0.9800
C3—C4	1.507 (2)	C14—H14C	0.9800
C3—H3A	0.9900	C15—H15A	0.9800
C3—H3B	0.9900	C15—H15B	0.9800
C4—C5	1.471 (2)	C15—H15C	0.9800
C4—C15	1.505 (3)	C16—C17	1.383 (3)
C5—C6	1.511 (2)	C16—C21	1.393 (2)
C5—H5A	1.0000	C17—N18	1.339 (2)
C6—C7	1.556 (2)	C17—H17A	0.9500
C6—H6A	1.0000	N18—C19	1.330 (2)
C7—C11	1.510 (2)	C19—N20	1.327 (3)
C7—C8	1.546 (2)	C19—H19A	0.9500
C7—H7A	1.0000	N20—C21	1.335 (3)
C8—C9	1.547 (2)	C21—H21A	0.9500
C8—H8A	0.9900		
			
C5—O1—C4	60.94 (10)	H8A—C8—H8B	107.8
C12—O2—C6	111.31 (12)	C10—C9—C8	113.70 (14)
C10—C1—C2	128.05 (17)	C10—C9—H9A	108.8
C10—C1—H1A	116.0	C8—C9—H9A	108.8
C2—C1—H1A	116.0	C10—C9—H9B	108.8
C1—C2—C3	110.73 (14)	C8—C9—H9B	108.8
C1—C2—H2A	109.5	H9A—C9—H9B	107.7
C3—C2—H2A	109.5	C1—C10—C14	124.58 (16)
C1—C2—H2B	109.5	C1—C10—C9	121.22 (16)
C3—C2—H2B	109.5	C14—C10—C9	114.20 (15)
H2A—C2—H2B	108.1	C13—C11—C12	119.20 (15)
C4—C3—C2	108.68 (14)	C13—C11—C7	132.19 (16)
C4—C3—H3A	110.0	C12—C11—C7	108.42 (13)
C2—C3—H3A	110.0	O3—C12—O2	121.43 (16)
C4—C3—H3B	110.0	O3—C12—C11	128.78 (16)
C2—C3—H3B	110.0	O2—C12—C11	109.75 (13)
H3A—C3—H3B	108.3	C11—C13—C16	127.89 (16)
O1—C4—C5	59.08 (10)	C11—C13—H13A	116.1
O1—C4—C15	112.05 (15)	C16—C13—H13A	116.1
C5—C4—C15	121.46 (15)	C10—C14—H14A	109.5
O1—C4—C3	118.08 (14)	C10—C14—H14B	109.5
C5—C4—C3	116.40 (15)	H14A—C14—H14B	109.5
C15—C4—C3	116.73 (15)	C10—C14—H14C	109.5
O1—C5—C4	59.97 (10)	H14A—C14—H14C	109.5
O1—C5—C6	120.41 (14)	H14B—C14—H14C	109.5
C4—C5—C6	122.16 (14)	C4—C15—H15A	109.5
O1—C5—H5A	114.5	C4—C15—H15B	109.5
C4—C5—H5A	114.5	H15A—C15—H15B	109.5
C6—C5—H5A	114.5	C4—C15—H15C	109.5
O2—C6—C5	109.48 (12)	H15A—C15—H15C	109.5
O2—C6—C7	106.97 (12)	H15B—C15—H15C	109.5
C5—C6—C7	112.07 (13)	C17—C16—C21	115.11 (16)
O2—C6—H6A	109.4	C17—C16—C13	124.52 (15)
C5—C6—H6A	109.4	C21—C16—C13	120.31 (15)
C7—C6—H6A	109.4	N18—C17—C16	123.19 (16)
C11—C7—C8	115.01 (13)	N18—C17—H17A	118.4
C11—C7—C6	102.45 (13)	C16—C17—H17A	118.4
C8—C7—C6	115.15 (13)	C19—N18—C17	115.52 (17)
C11—C7—H7A	107.9	N20—C19—N18	127.34 (18)
C8—C7—H7A	107.9	N20—C19—H19A	116.3
C6—C7—H7A	107.9	N18—C19—H19A	116.3
C7—C8—C9	112.99 (13)	C19—N20—C21	115.48 (15)
C7—C8—H8A	109.0	N20—C21—C16	123.23 (17)
C9—C8—H8A	109.0	N20—C21—H21A	118.4
C7—C8—H8B	109.0	C16—C21—H21A	118.4
C9—C8—H8B	109.0		
			
C10—C1—C2—C3	−110.99 (19)	C2—C1—C10—C14	−8.9 (3)
C1—C2—C3—C4	53.54 (19)	C2—C1—C10—C9	171.47 (16)
C5—O1—C4—C15	−114.44 (16)	C8—C9—C10—C1	−107.23 (18)
C5—O1—C4—C3	105.54 (17)	C8—C9—C10—C14	73.07 (19)
C2—C3—C4—O1	−152.28 (15)	C8—C7—C11—C13	−56.5 (2)
C2—C3—C4—C5	−84.95 (18)	C6—C7—C11—C13	177.78 (17)
C2—C3—C4—C15	69.54 (18)	C8—C7—C11—C12	128.73 (15)
C4—O1—C5—C6	111.94 (17)	C6—C7—C11—C12	3.06 (16)
C15—C4—C5—O1	98.43 (17)	C6—O2—C12—O3	172.90 (16)
C3—C4—C5—O1	−108.37 (16)	C6—O2—C12—C11	−9.33 (18)
O1—C4—C5—C6	−109.10 (17)	C13—C11—C12—O3	5.6 (3)
C15—C4—C5—C6	−10.7 (2)	C7—C11—C12—O3	−178.86 (18)
C3—C4—C5—C6	142.53 (15)	C13—C11—C12—O2	−171.94 (15)
C12—O2—C6—C5	132.85 (14)	C7—C11—C12—O2	3.59 (18)
C12—O2—C6—C7	11.21 (17)	C12—C11—C13—C16	172.57 (15)
O1—C5—C6—O2	36.7 (2)	C7—C11—C13—C16	−1.7 (3)
C4—C5—C6—O2	108.24 (17)	C11—C13—C16—C17	−29.8 (3)
O1—C5—C6—C7	155.20 (14)	C11—C13—C16—C21	153.17 (17)
C4—C5—C6—C7	−133.25 (15)	C21—C16—C17—N18	−3.7 (3)
O2—C6—C7—C11	−8.19 (16)	C13—C16—C17—N18	179.10 (17)
C5—C6—C7—C11	−128.18 (14)	C16—C17—N18—C19	1.1 (3)
O2—C6—C7—C8	−133.78 (14)	C17—N18—C19—N20	1.7 (3)
C5—C6—C7—C8	106.23 (15)	N18—C19—N20—C21	−1.4 (3)
C11—C7—C8—C9	153.12 (15)	C19—N20—C21—C16	−1.7 (3)
C6—C7—C8—C9	−88.08 (18)	C17—C16—C21—N20	4.0 (2)
C7—C8—C9—C10	69.71 (19)	C13—C16—C21—N20	−178.66 (16)
